# Phase II study of neoadjuvant therapy with nab-paclitaxel and cisplatin followed by surgery in patients with locally advanced esophageal squamous cell carcinoma

**DOI:** 10.18632/oncotarget.9562

**Published:** 2016-05-23

**Authors:** Yun Fan, Youhua Jiang, Xinming Zhou, Qixun Chen, Zhiyu Huang, Yanjun Xu, Lei Gong, Haifeng Yu, Haiyan Yang, Jinshi Liu, Tao Lei, Qiang Zhao, Weimin Mao

**Affiliations:** ^1^ Department of Chemotherapy, Zhejiang Cancer Hospital, Hangzhou, China; ^2^ Key Laboratory Diagnosis and Treatment Technology on Thoracic Oncology and Cancer Research Institute, Hangzhou, China; ^3^ Department of Thoracic Surgery, Zhejiang Cancer Hospital, Hangzhou, China

**Keywords:** esophageal squamous cell carcinoma, nab-paclitaxel, cisplatin, neoadjuvant chemotherapy, esophagectomy

## Abstract

**Background:**

We carried out a phase II study to evaluate the efficiency and safety of the combination of nanoparticle albumin bound-paclitaxel (nab-paclitaxel) and cisplatin as preoperative chemotherapy for locally advanced esophageal squamous cell carcinoma (ESCC)

**Results:**

From Oct 2011 to Dec 2012, 35 patients were enrolled and received neoadjuvant chemotherapy. Thirty patients underwent surgery and achieved a 100% R0 resection. Pathological complete response (pCR) rate was 13.3% and near pCR rate was 6.7%. Down-staging was achieved in 19 patients. With median follow-up of 37.8 months, 16 patients were still alive. One-, 2- and 3- year overall survival (OS) rate was 90.0%, 70.0% and 43.3%, respectively. This treatment resulted in a median disease-free survival (DFS) of 34.7 months and a median OS of 37.8 months. Median DFS and OS of down-staged patients were significantly longer than those of non-downstaged patients. The grade 4 toxicities during neoadjuvant chemotherapy were limited to neutropenia (2.9%) and vomiting (2.9%).

**Methods:**

Patients with locally advanced ESCC (stage IIA to IIIC) and performance status 0-1 were enrolled and received two cycles of nab-paclitaxel (100 mg/m^2^) on day 1, 8, 22 and 29, and cisplatin (75 mg/m^2^) on day 1 and 22, followed by resection. Two cycles of adjuvant chemotherapy with the same regimen were given. Postoperative radiotherapy was permitted and decided by radiation therapist.

**Conclusion:**

Weekly nab-paclitaxel with three-weekly cisplatin seems effective and safe as a neoadjuvant chemotherapy strategy for locally advanced ESCC. Down-staged patients have favorable outcome.

**ClinicalTrials.gov Identifier:**

NCT01258192

## INTRODUCTION

Esophageal cancer is a malignant tumor with a poor prognosis that is common in China [1]. According to the 2014 Chinese cancer registry annual report, the incidence of esophageal carcinoma was 22.87 per 10 million, ranking fifth; the annual mortality rate was 17.35 per 10 million, ranking forth [2]. With surgery alone, the 5-year survival rate for T2-T3N0 disease is < 30-40%, and it declines to < 25% with nodal involvement [3]. Despite substantial advances in the multidisciplinary therapy for locally advanced resectable esophageal cancer, prognosis remains poor.

Many clinical studies had explored the efficacy and safety of neoadjuvant therapy for locally advanced esophageal carcinoma. Some of them reported positive results, which suggest that neoadjuvant therapy, chemotherapy alone or combined with radiotherapy, can benefit patients and improve prognosis [4-8]. Although part of researchers prefer preoperative chemoradiation in esophageal cancer, neoadjuvant chemotherapy seems much safer with a reduced postoperative mortality [9-10]. Moreover, although preoperative chemoradiation is a standard strategy in the treatment of locally advanced esophageal cancer in USA, an updated meta-analysis shows no clear advantage of neoadjuvant chemoradiation over neoadjuvant chemotherapy alone [11]. Of note, when limited to the patients with ESCC included in that meta-analysis (65% of patients with SCC), neoadjuvant chemoradiation significantly increased survival versus surgery alone but not versus neoadjuvant chemotherapy [11]. Thus, the value of preoperative chemotherapy still needs further substantiation.

Even if trials often had been designed to evaluate the survival benefits from cisplatin and 5-fluorouracil (FU) as a preoperative chemotherapy scheme [10, 12-13] the best neoadjuvant chemotherapy regimen has not been well established. Nab-Paclitaxel (nab-PC) is a new generation formulation of paclitaxel. It is a solvent-free albumin-bound form of paclitaxel [14-16]. When compared with solvent-based-paclitaxel (sb-PC), nab-PC has various advantages, including the ability to deliver higher doses of paclitaxel over a shorter infusion time (30 minutes vs 3 hours for sb-PC) and the elimination of pre-medications to pre-vent hypersensitivity reaction. Other advantages of nab-PC over sb-PC include enhanced transport of paclitaxel across endothelial cells and greater delivery of paclitaxel to tumors. In a preclinical study, more than fourfold nab-PC was transported across endothelial cells than sb-PC [16]. A phase III trial compared the effects of weekly nab-PC versus sb-PC in combination with carboplatin for first-line treatment of non-small cell lung cancer (NSCLC). Weekly nab-PC outclassed sb-PC resulting in a significantly improved overall response rate (ORR) in advanced NSCLC [17]. Moreover, the combination of nab-PC and cisplatin had already been realized as a highly effective and well-tolerated first-line treatment in metastatic ESCC [18]. Based on the hot issues in locally advanced esophageal cancer therapy, we carried out a phase II clinical study to determine the pathological response rate, efficacy and safety of preoperative neoadjuvant chemotherapy with weekly nab-PC and cisplatin in treating locally advanced ESCC.

## RESULTS

**Figure 1:**
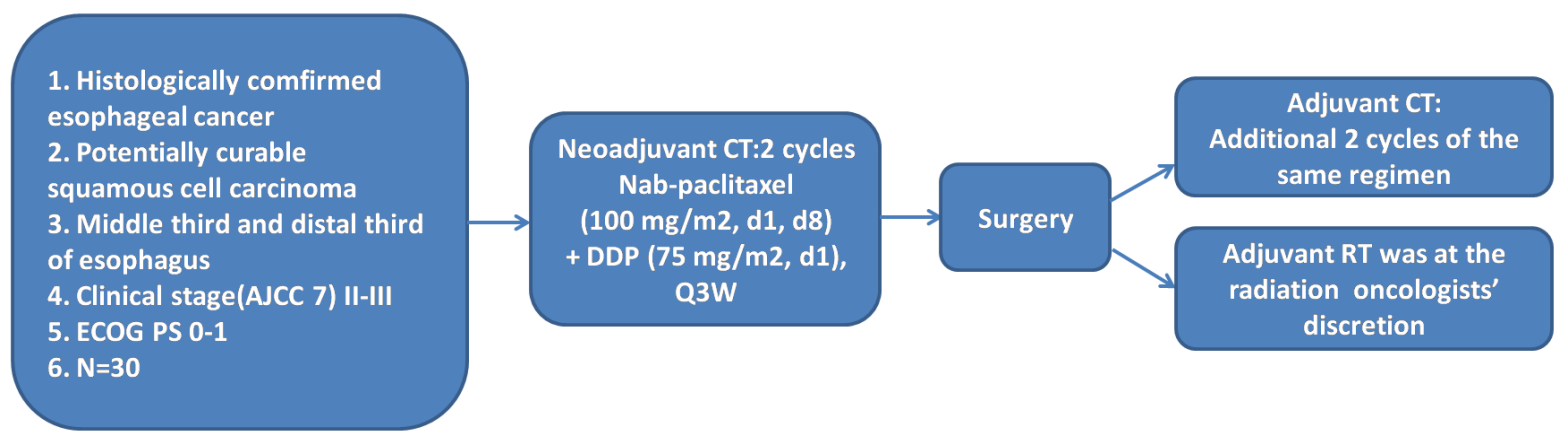
Study design All patients had biopsy-proven resectable locally advanced squamous cell carcinoma of the middle third and distal third of esophagus and an ECOG Performance Status 0-1. All eligible patients received nab-PC (100 mg/m2, d1, d8, d22 and d29) and cisplatin (75 mg/m2, d1 and d22) as neoadjuvant chemotherapy, followed by esophagectomy and adjuvant chemotherapy with or without radiotherapy.

**Figure 2:**
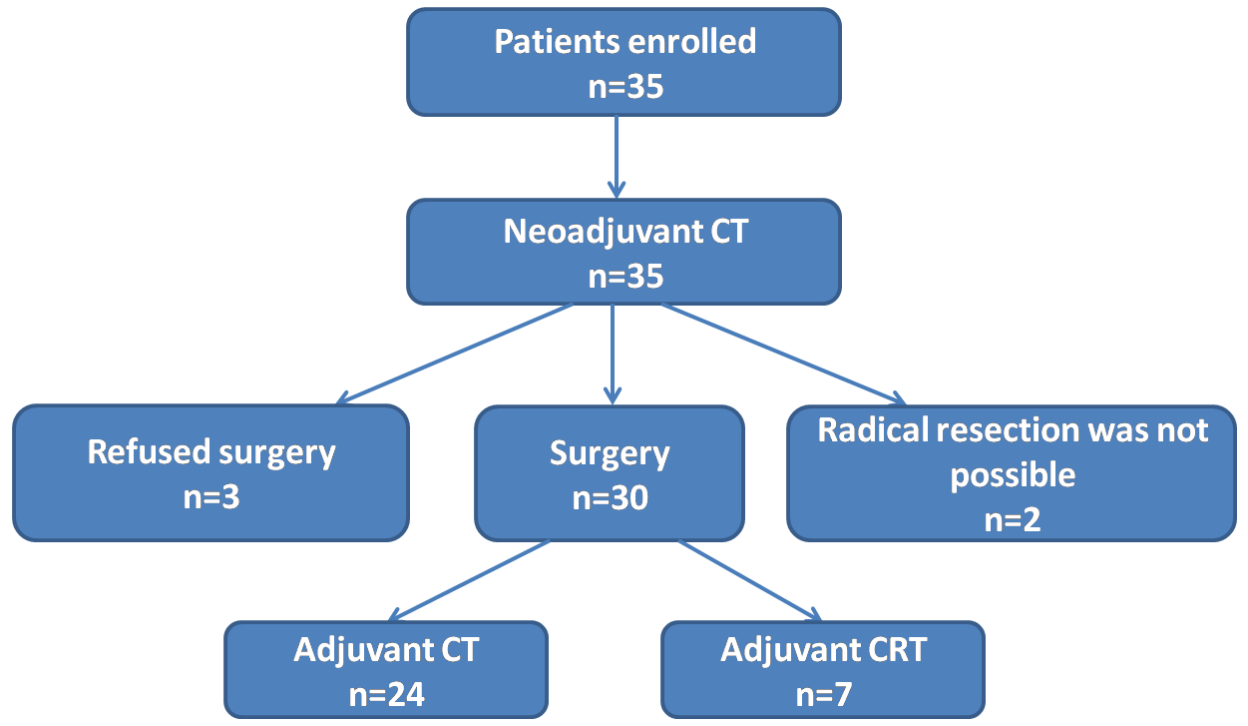
Study scheme Total planned doses of nab-PC and cisplatin were administered in 35 patients. Five patients did not receive surgery after neoadjuvant chemotherapy, among them 2 suffered progressive disease, 3 refused surgery. Twenty-four patients accepted two cycles of adjuvant chemotherapy (CT) with the same regimen after surgery, and 7 patients received adjuvant radiotherapy after adjuvant chemotherapy (CRT).

From Oct 2011 to April 2012, 10 patients were enrolled and received 2 cycles of neoadjuvant chemotherapy with nab-PC and cisplatin. They all subsequently underwent surgery. The enrollment was continued when there were 20% (2/10) pCR. Finally, 35 patients were enrolled until Dec 2012 at Zhejiang Cancer Hospital, including 31 males and 4 females, with a median age of 59 year (48-70 year) (Table 1). All patients had biopsy-proven resectable locally advanced squamous cell carcinoma of the middle third and distal third of esophagus and an Eastern Cooperative Oncology Group (ECOG) Performance Status 0-1 (Figure 1). Stage II A, II B, III A, III B and III C disease was found in 3 (8.6%), 5 (14.3%), 10 (28.6%), 8 (22.9%) and 9 (25.7%) patients, respectively. The clinical characteristics of patients are listed in Table 1. All 35 patients have completed the two cycles of neoadjuvant chemotherapy. Thirty patients (30/35, 85.7%) underwent surgery after 2 cycles of neoadjuvant chemotherapy (Figure 2).

**Table:** The clinical characteristics of patients enrolled (N = 35)

Characteristics	Patients, *N* (%)
**Sex**	
** male**	31 (88.6)
** female**	4 (11.4)
**Age, years**	
**median**	59
**Range**	48-70
**Tumor location**	
**Middle third**	30 (85.7)
**Distal third**	5 (14.3)
**ECOG performance status**	
**0**	25 (71.4)
**1**	10 (28.6)
**Clinical T stage**	
**T1**	0 (0)
** T2**	9 (25.7)
** T3**	20 (57.1)
** T4**	6 (17.1)
**Clinical N stage**	
**N0**	7 (20)
**N1**	10 (28.6)
**N2**	13 (37.1)
**N3**	5 (17.3)
**Clinical stage**	
**IIA**	3 (8.6)
**IIB**	5 (14.3)
**IIIA**	10 (28.6)
**IIIB**	8 (22.9)
**IIIC**	9 (25.7)

ECOG, Eastern Cooperative Oncology Group.

### Treatment

Total planned doses of nab-PC and cisplatin were administered in 100% of patients. Five patients did not receive surgery after neoadjuvant chemotherapy, among them 2 suffered progressive disease and were unable to undergo radical resection, 3 refused surgery. Twenty-four patients (24/30, 80.0%) accepted two cycles of adjuvant chemotherapy with the same regimen after surgery, and among them, 7 patients (7/30, 23.3%) received adjuvant radiotherapy after adjuvant chemotherapy (Figure 2).

### Efficacy of neoadjuvant chemotherapy

The ORR (according to RECIST version 1.1) after completion of 2 cycles of neoadjuvant chemotherapy with nab-PC and cisplatin amounted to 65.7% (complete response (CR): 6/35, partial response (PR): 17/35) as evaluated by enhancement thoracoabdominal CT scan.

### Histopathologic analysis and tumor down-staging

All of the 30 patients who underwent surgery had R0 resection (100%). pCR was achieved in 4 patients (4/30, 13.3%) and near pCR (defined as microfoci of tumor cells on the primary tumor without lymph nodal metastasis) in 2 patients (2/30, 6.7%). Significant down-staging was observed in 19 patients (19/30, 63.3%) (Table 2).

**Table:** Histopathologic analysis and tumor down-staging

	N (*N* = 30,%)		N(*N* = 35%)
**pCR**	4 (13.3)	CR	6(17.1)
**Near pCR**	2 (6.7)	PR	17(48.6)
**R0 resection rate**	30 (100)	PD	2(5.7)
**Down-staging rate**	19 (63.3)	ORR	33(65.7)

pCR: pathologic complete response

Near pCR: microfoci of tumor cells on the primary tumor without lymph nodal metastasis

R0: the resection was defined as curative

CR: complete response

### Survival

**Figure 3:**
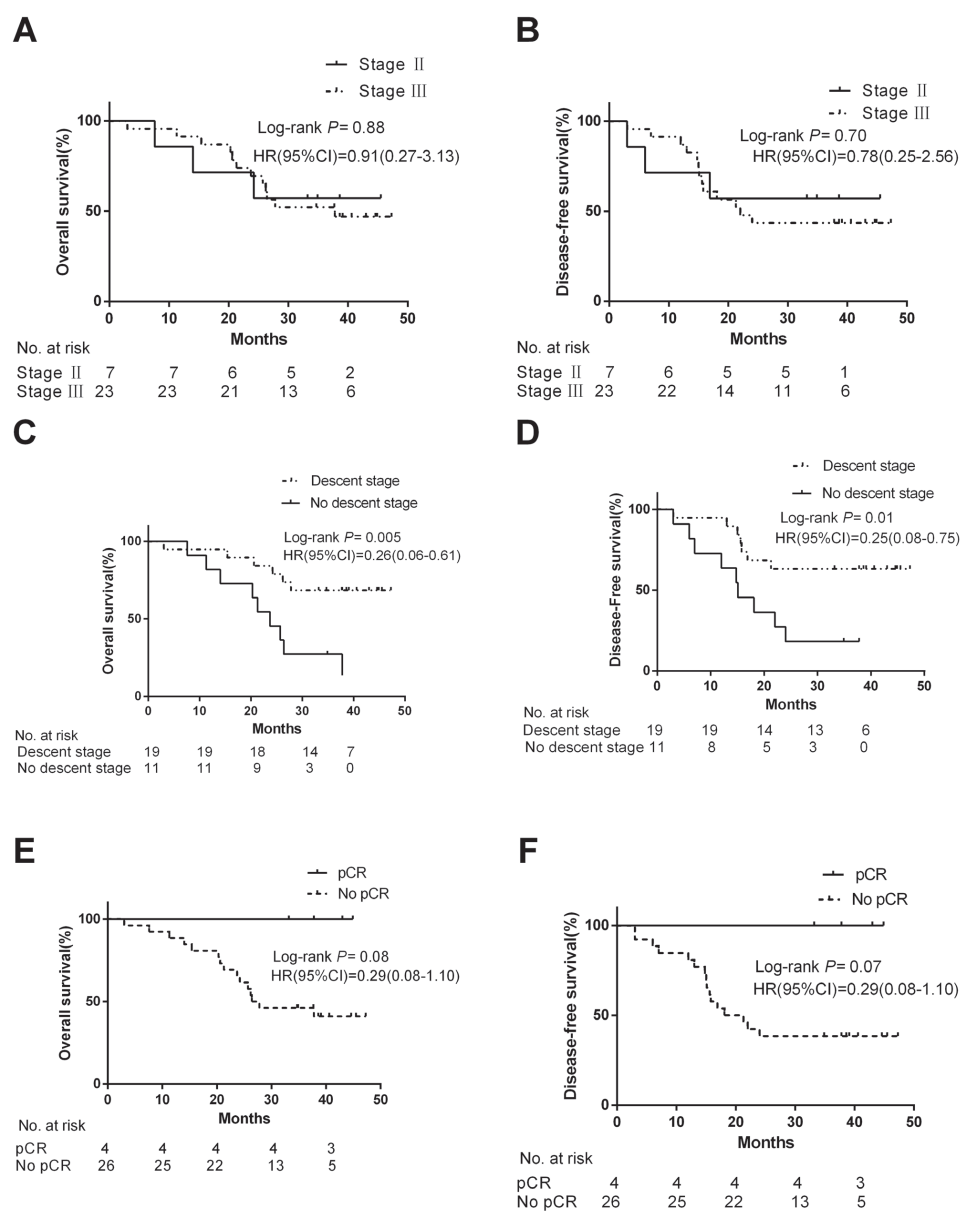
Kaplan-Meier analysis of progression-free survival (PFS) and overall survival (OS) of patients in different groups Figure 3 **A.**-**B.** OS and DFS were not significantly different between patients with stage II and stage III disease. Figure 3 **C.**-**D.** Median OS and DFS of descent stage patients were significantly longer than of no descent stage patients. Figure 3 **E.**-**F.** Median OS and DFS were longer in pCR patients than in non-pCR patients but that difference did not reach significance.

The entire 30 patients who underwent surgery were followed up. Median follow-up was 37.8 months. Total number of deaths was 14 (46.7%) while 16 (50.4%) patients were still alive. Moreover, 14 patients were alive without disease relapse. One-, 2- and 3-y OS rates were 90.0%, 70.0% and 43.3%, respectively, and 1-, 2- and 3- y DFS rates were 83.3%, 46.7% and 40.0%, respectively. The treatment resulted in a median DFS of 34.7 months and a median OS of 37.8 months. OS and DFS were not significantly different between patients with stage II and stage III disease (Figure 3A-3B). Median OS and DFS of down-staged (descent stage) patients were significantly longer than of non-downstaged (no descent stage) patients (HR: 0.26, 95% CI: 0.06- 0.61; *P* = 0.005 and HR: 0.25, 95% CI: 0.08-0.75; *P* = 0.01, respectively) (Figure 3C-3D). Median OS and DFS were longer in pCR patients than in non-pCR patients but that difference did not reach significance (*P* = 0.08 and *P* = 0.07, respectively) (Figure 3E-3F).

### Adverse events

All patients were assessable for treatment-related toxicities. Adverse events (AEs) are summarized in Table 3. The G3 toxicities occurring in the course of neoadjuvant chemotherapy included neutropenia (3/35, 8.6%), anemia (3/35, 8.6%), thrombocytopenia (2/35, 5.7%), febrile neutropenia (3/35, 8.6%), nausea (2/35, 5.7%), as well as vomiting (4/35, 11.4%). The G4 toxicities only consisted of neutropenia (1/35, 2.9%) and vomiting (1/35, 2.9%) (Table 3). No treatment-related mortality was observed before or after surgery. No dosage reductions were necessary during the neoadjuvant period. Complications associated with surgery included one case of anastomotic leak (1/30, 3.3%). During the adjuvant chemotherapy period, G3 and G4 hematologic AEs were observed in 5 (5/24, 20.8%) patients, in whom the dose of nab-PC was reduced by 20%.

**Table:** Toxicities in 35 evaluable patients during neoadjuvant chemotherapy

Adverse events	G1-G4 (%)	G3, *N* (%)	G4, *N* (%)
**Hematologic**			
Anemia	21 (60)	3 (8.6)	0 (0)
Neutropenia	25 (71.4)	3 (8.6)	1 (2.9)
Thrombocytopenia	6 (17.1)	2 (5.7)	0 (0)
**Nonhematologic**			
Febrile neutropenia	5 (14.3)	3 (8.6)	0 (0)
Fatigue	15 (42.9)	0 (0)	0 (0)
Nausea	28 (80)	2 (5.7)	0 (0)
Vomiting	19 (54.3)	4 (11.4)	1 (2.9)
Diarrhea	1 (2.9)	0 (0)	0 (0)
Alopecia	10 (28.6)	0 (0)	0 (0)
Sensory neuropathy	6 (17.1)	0 (0)	0 (0)
ALT	2 (5.7)	0 (0)	0 (0)

## PATIENTS AND METHODS

### Eligibility

Treated patients met the following inclusion criteria: (I) age ranges from 18 to 70 years, (II) ECOG Performance Status 0-1, (III) biopsy-proven resectable locally advanced thoracic ESCC, (IV) untreated clinical stage IIA to IIIC as assessed by enhancement computed tomography (CT) scan and/or positron tomography (PET/CT) scan, and endoscopic ultrasound (EUS), (V) tumor location with middle third and distal third of esophagus, (VI) adequate bone marrow, hepatic and renal function, (VII) non-pregnant and non-breast feeding, (VIII) no prior invasive malignancy, (IX) not any prior anticancer therapy, and, (X) tumor can be measured according to RECIST criteria. All patients provided written informed consent. Ethical committee approval was given, and the trial was registered on the ClinicalTrials.gov Web site (NCT01258192). Exclusion reasons comprise: (I) carcinoma at the upper part of esophagus, (II) histologic diagnosis of adenocarcinoma of esophagus, (III) prior treatment for esophageal cancer, (IV) active infection, (V) pregnant or breast feeding, (VI) history of significant neurological or mental disorder, including seizures or dementia, and, (VII) prior invasive malignancy in previous 5 years (except for carcinoma in situ).

### Pretreatment tests and staging

Pretreatment clinical tests include history, clinical examination, routine laboratory blood tests, endoscopy with biopsy, bronchoscopy, respiratory function tests, and ECG. Staging was performed by enhancement thoracoabdominal CT scan and/or PET/CT scan and EUS based on the 7th Union for International Cancer Control TNM classification [19]. Cervical ultrasound and radionucleotide bone scan were optional.

### Treatments

Enrollment was performed by the clinicians and all eligible patients received nab-PC (100 mg/m^2^, d1, d8, d22 and d29) and cisplatin (75 mg/m^2^, d1 and d22) as neoadjuvant chemotherapy (Figure 1), followed by esophagectomy and adjuvant chemotherapy with or without radiotherapy.

Neoadjuvant chemotherapy: Neoadjuvant chemotherapy was composed of two cycles of nab-PC and cisplatin. All chemotherapy agents were dosed based on actual body surface area. Nab-PC 100 mg/m^2^ was delivered by intravenous infusion on day 1, 8, 22 and 29. Cisplatin 75mg/m^2^ was delivered by intravenous infusion on day 1 and 22. Prophylactic use of granulocyte colony stimulating factor (G-CSF) before neoadjuvant chemotherapy was not allowed.

Surgery: All enrolled patients underwent clinical re-evaluation 3 weeks (day43) after termination of the second cycle of neoadjuvant chemotherapy, including physical examination, weight evaluation, blood laboratory analysis, and thoracoabdominal enhancement CT scan. If there was no evidence of metastatic disease, curative resection was carried out. Surgery was performed approximately 4 to 8 weeks after completion of neoadjuvant chemotherapy. The surgical procedure was selected by the surgeon according to tumor site, tumor stage and local practice. Esophagectomy approaches were transthoracic, transhiatal, or thoracoabdominal. Esophagectomy methods applied in this study include Ivor-Lewis and McKeown. Overlying pleura and adjacent soft tissues were included to ensure adequate radial margins. Nodal staging was recommended.

Postoperation treatment: Two cycles of adjuvant chemotherapy with the same regimen (nab-PC and cisplatin) were given starting 4-6 weeks after the resection. Postoperative three-dimensional conformal radiotherapy was permitted and was selected by the radiation therapist according to tumor stage. Radiotherapy was given after 2 cycles of adjuvant chemotherapy. The dose and fractionation regimen was either 50.4 Gy in 28 fractions or abiologically equivalent dose.

### Dose modifications

If disease progression or new metastasis occurred in the course of neoadjuvant chemotherapy, the second cycle was not permitted and immediate surgery was mandated. Dose modification was carried out for agents based on their estimated causal relationship to the toxicity. Doses of nab-PC and cisplatin were reduced by 20% in the second cycle if grade 4 neutropenia, anemia, or thrombocytopenia was observed. The dose of cisplatin was reduced by 20% in the second cycle if creatinine clearance (CCr) was 50 ≤ CCr < 60, by 40% if CCr was 40 ≤ CCr < 50, and stopped if CCr was < 40 mL/min, or ototoxicity of grade 2 or higher was observed.

### Pathologic analysis

The histopathological investigations included hematoxylin and eosin (HE) staining and were performed by two professional pathologists independently of each other. Histopathologic examination indicated whether the resection was defined as curative (R0) or whether there was residual microscopic disease (R1) or macroscopic tumor (R2). Pathologic response to the neoadjuvant chemotherapy was defined by tumor regression grade according to the Mandard classification [20].

### Study endpoints

The primary study endpoint was pCR. The secondary endpoints included R0 resection rate, down-staging rate, effectiveness, safety, 3-year overall survival (3-y OS) and 3-year disease-free survival (3-y DFS). pCR was defined as no viable residual tumor cells. Near pCR was defined as ≤ 10% residual carcinoma in patients who did not qualify as pCR [21]. OS was recorded from the initiation of recruitment to the date of death or that of the last follow-up visit. DFS were calculated from the date of recruitment to documented progression or the date of death. Tumor response was evaluated for patients who had measurable lesions according to RECIST version 1.1.

### Follow-up

Patients were followed-up every 3 months during the first 2 years after date of recruitment, and every 6 months from the third year onwards. Disease recurrence was defined as locoregional (esophageal bed or anastomotic or regional lymph nodes) or metastatic (supraclavicular lymphnodes or distant organs). A clinical examination and thoracoabdominal enhancement CT were performed every time. Follow-up was complete for all the 30 patients who underwent surgery, with a median 37.8 months of follow-up.

### Statistical analysis

The sample size was determined by the Simon two-stage design method. The study was continued when there were at least 20% (2/10) pCR at stage 1. Then the study proceeded to the second stage with a total sample size of 30 patients. Our report reflects the final analysis after long-term follow-up. The Kaplan-Meier method was used. Corresponding HRs were calculated with 95% CIs using the Cox proportional hazards model. The Cox proportional model was used to evaluate various prognostic factors. Analyses were conducted using the computer software SPSS version 17.0 (SPSS Inc., Chicago, IL, USA).

### Adverse events

AEs were graded using CTCAE Version 4.0. AEs were recorded from the day of enrollment to 30 days following the accomplishment of treatments and were summarized as frequency and percent. When a patient suffered a particular AE event several times, only the highest grade was considered. A stopping rule was included for AE in this trial. If 2 of the first 10 patients or if 30% or more patients suffered grade ≥ 4 non-hematologic AEs that were possibly related to treatment, the team was required to review the data before proceeding patient recruitment further.

## DISCUSSION

Our trial explored the benefits and safety of weekly nab-PC and cisplatin chemotherapy as a neoadjuvant strategy for locally advanced resectable ESCC, including 77.1% (27/35) stage III patients. The approach not only resulted in a 100% R0 resection rate, and a 63.3% (19/30) down-staging rate, but also came at a satisfactory OS and DFS rate with tolerable toxicity. The response rate observed in our study was encouraging, with a pCR plus near pCR rate of 20% (13.3% plus 6.7%). That 13.3% (4/30) pCR rate exceeds the 0%-7.7% pCR rates reported for other cisplatin-based regimens [22-24].

Firstly, the most important result from our study was that a 63.3% down-staging rate was achieved with neoadjuvant combination treatment with weekly nab-PC and cisplatin, and down-staged patients showed significantly longer survival than patients without down-staging. The correlation between down-staging and histological response to any preoperative therapy and prognosis has been reported previously in ESCC [7, 25]. This finding was in according with other studies indicating that down-staging with preoperative therapy could result in achieving a high tumor response and improving the complete resection rate and prolonging survival. That 63.3% down-staging rate exceeds the down-staging rates reported for other cisplatin-based regimens. Thus, this encouraging result indicates that evaluation of nab-PC and cisplatin as neoadjuvant chemotherapy regimens in randomized trials seems to be warranted.

There was also a significant relationship between pCR and DFS/OS. Our findings are in line with other studies demonstrating that achievement of a pCR is associated with better DFS and OS [26-28]. For instance, the RTOG trial 8911 showed that patients who achieved a pCR following preoperative therapy had a significantly improved DFS when compared with non-pCR patients [25]. Both RTOG 8911 and OEO2, another large trial of preoperative chemotherapy in resectable esophageal cancer, have also shown that unless patients did undergo R0 resection their overall survival remained very poor [25-28]. These results also imply that a major effect of neoadjuvant chemotherapy is to reduce tumor volume thereby increasing the potential for R0 resection.

With a median follow-up of 37.8 months, in our study 16/30 (50.4%) patients were still alive and 14/16 (87.5%) patients were alive without disease relapse. One-, 2- and 3-year OS rates were 90.0%, 70.0% and 43.3% respectively. By contrast, the INT0113 study, which comprised 49% patients with ESCC, showed no difference in OS between the neoadjuvant chemotherapy group and the surgery alone group [12]. First results from the British MRC study (33% ESCC) published in 2002 showed that neoadjuvant chemotherapy with cisplatin and 5-FU increased the 2-year survival rate from 34% to 43%, and the R0 resection rate from 54% to 60% [28]. The updated survival data from that study further suggested that the 5-year survival rate was 23% in the neoadjuvant chemotherapy group, being significantly higher than 17.1% in the surgery group [13]. In our study the R0 resection rate reached 100% and the 2-year OS rate increased to 69.8%, both being obviously better than in INT0113 and the MRC study [12, 13]. It therefore seems that weekly nab-PC and cisplatin as a neoadjuvant chemotherapy for locally advanced ESCC might be a promising alternative. Moreover, when compared with results from a phase II study of concurrent chemoradiotherapy for inoperable ESCC [29], our 1-year (90% *vs* 75%), 2-year (70% *vs* 54%) and 3-year survival rates (43.3% *vs* 41%) seem encouraging.

In order to demonstrate the value of neoadjuvant chemotherapy for locally advanced ESCC, several trials were also run one after another in Japan [30-32]. The JCOG9907 study on resectable stage II/III ESCC demonstrated that the 5-year OS was significantly improved by neoadjuvant chemotherapy consisting of two courses of cisplatin plus 5-fluorouracil compared with postoperative chemotherapy (55%*vs*43%,*P* = 0.04). Consequently, the standard of care for locally advanced thoracic esophageal cancer in Japan has changed from postoperative to preoperative chemotherapy [32]. When focusing on patients with stage III disease, which constitute a higher percentage in our study than in the JCOG9907 study (77.1% *vs*51%), our 3-year OS rate was 50.4% and the 5-year OS has not been reached yet.

Besides provisional evidence of superior efficacy, the toxicity profile of weekly nab-PC plus cisplatin was also favorable. In the study, 100% patients completed 2 cycles of neoadjuvant chemotherapy without dosage reduction. Compared to a phase II study of concurrent chemoradiotherapy using sb-PC plus cisplatin for inoperable ESCC [29], grade 3 and 4 neutropenia was lower (11.5% *vs* 61.9%). During the adjuvant chemotherapy period, grade 3 and 4 hematologic AEs were observed only in 5 (20.8%) patients. Triple-modality therapy, including radiotherapy, chemotherapy and surgery, is widely considered to be a standard treatment for locally advanced esophageal cancer [29, 33-40]. It seems, however, that concurrent chemoradiation can be accompanied with more severe toxicities and a greater risk of operative mortality [10, 41-44]. For instance, in the CROSS study (including 23% squamous cell carcinoma) [33], anastomotic leak occurred in 36 (22%) patients and 10 (6%) deaths came up after surgery in the chemoradiotherapy and surgery group. In the Stahl study, operative mortality is raised to 10.2% after chemoradiotherapy *vs* 3.8% in surgery alone group [45]. In our study, no treatment-related mortality was observed before or after surgery, and complications associated with surgery consisted of only one case of anastomotic leak (3.3%). Some mature data, take JCOG9907 and OEO2 trials for example, confirm survival advantages for patients with locally advanced resectable esophageal cancer treated by preoperative chemotherapy with a non-elevated risk of complications or postoperative mortality [13, 28, 46-48], which is consistent with our results. Thus, our data show that the two-drug-based regimen of nab-PC and cisplatin as a neoadjuvant chemotherapy is safe and feasible in locally advanced ESCC.

To the best of our knowledge, this clinical trial seems to be the first one evaluating the efficiency and safety of the combination of nab-PC and cisplatin as preoperative chemotherapy for the treatment of resectable ESCC. However, we have to admit that when compared with other similar clinical researches, the biggest weakness in this study is that the sample size is rather small. Moreover, with a short median follow-up period, the study so far provides no sound overall survival data at 5 years.

In conclusion, our data demonstrate that a combination regimen of nab-PC and cisplatin as neoadjuvant chemotherapy shows high activity in terms of 100% R0 resection, 63.3% down-staging rate and 13.3% pCR rate, and is safe in patients with locally advanced ESCC. Moreover, our data also indicate that the achievement of a pCR to neoadjuvant weekly nab-paclitaxel plus cisplatin chemotherapy might serve as a predictor of favorable outcome. Thus, evaluation of nab-PC and cisplatin in randomized trials seems to be warranted.
